# Influenza A(H7N9) Virus Antibody Responses in Survivors 1 Year after Infection, China, 2017

**DOI:** 10.3201/eid2404.171995

**Published:** 2018-04

**Authors:** Mai-Juan Ma, Cheng Liu, Meng-Na Wu, Teng Zhao, Guo-Lin Wang, Yang Yang, Hong-Jing Gu, Peng-Wei Cui, Yuan-Yuan Pang, Ya-Yun Tan, Hui Hang, Bao Lin, Jiang-Chun Qin, Li-Qun Fang, Wu-Chun Cao, Li-Ling Cheng

**Affiliations:** Beijing Institute of Microbiology and Epidemiology, Beijing, China (M.-J. Ma, M.-N. Wu, T. Zhao, G.-L. Wang, H.-J. Gu, L.-Q. Fang, W.-C. Cao);; University of Florida, Gainesville, Florida, USA (Y. Yang);; Suzhou Municipal Center for Disease Control and Prevention, Suzhou, China (C. Liu, P.-W. Cui, Y.-Y. Pang, Y.-Y. Tan, H. Hang, B. Lin, J.-C. Qin, L.-L. Chen)

**Keywords:** H7N9 viruses, cohort, survivors, antibody response, serological, persistence, influenza, China, viruses, influenza A(H7N9)

## Abstract

Avian influenza A(H7N9) virus has caused 5 epidemic waves in China since its emergence in 2013. We investigated the dynamic changes of antibody response to this virus over 1 year postinfection in 25 patients in Suzhou City, Jiangsu Province, China, who had laboratory-confirmed infections during the fifth epidemic wave, October 1, 2016–February 14, 2017. Most survivors had relatively robust antibody responses that decreased but remained detectable at 1 year. Antibody response was variable; several survivors had low or undetectable antibody titers. Hemagglutination inhibition titer was >1:40 for <40% of the survivors. Measured in vitro in infected mice, hemagglutination inhibition titer predicted serum protective ability. Our findings provide a helpful serologic guideline for identifying subclinical infections and for developing effective vaccines and therapeutics to counter H7N9 virus infections.

The novel avian influenza A(H7N9) virus has caused 5 epidemic waves in China since its emergence in 2013. As of September 20, 2017, a total of 1,561 human cases were reported, with a case fatality rate of ≈39% (*1*). In particular, a substantial increase of 758 human cases was reported during the fifth epidemic, compared with the earlier 4 (*1*). Highly pathogenic H7N9 viruses emerged and infected both humans (*1*) and poultry (*2*) during the fifth epidemic. In addition, H7N9 virus readily obtained the 627K or 701N mutation in its polymerase basic (PB) 2 segment upon replication in ferrets (*3*), suggesting that the virus has pandemic potential and continues to pose grave risks to public health.

The H7N9 subtype has the highest risk score among the 12 novel influenza A viruses evaluated by the Centers for Disease Control and Prevention using the Influenza Risk Assessment Tool and is characterized as posing moderate- to high-potential pandemic risk (*4*). Apart from the ongoing monitoring of virologic and molecular characteristics of H7N9 viruses in poultry and humans, studies on the dynamic changes of antibody response in survivors are critical for serologic diagnosis, population-based seroepidemiologic surveys, and vaccine design and development. A few studies have investigated virus-specific antibody kinetics to the H7N9 virus in patients and their relationship with disease severity (*5*–*7*), but these studies were restricted to antibodies measured in the acute and convalescent phases. No follow-up studies have been done on dynamic antibody changes in survivors who had recovered from the disease. As a result, the long-term serologic response to H7N9 virus infections is poorly understood and remains of clinical interest. In our study, we investigated the long-term dynamic changes in antibody response in H7N9 survivors identified during the fifth epidemic in China and examined the relationship between antibody responses and clinical characteristics.

## Materials and Methods

### Study Design and Participants

**Figure 1:**
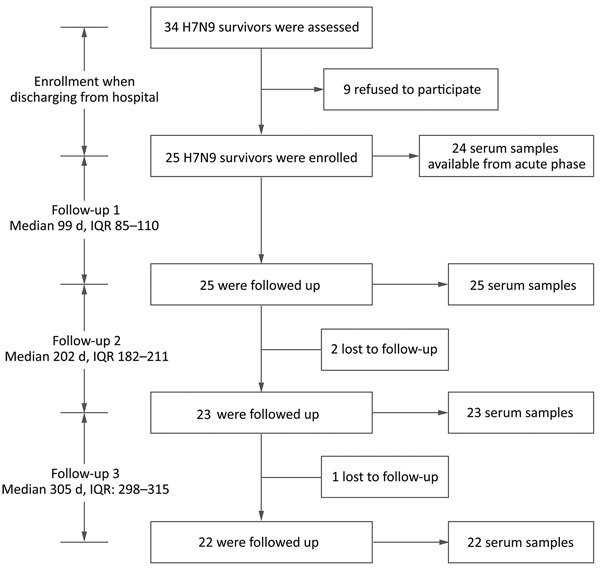
Schematic outline for study of influenza A(H7N9) virus antibody responses in survivors 1 year after infection, China, 2017. IQR, interquartile range.

During the fifth epidemic wave of the H7N9 virus (October 1, 2016–February 14, 2017), we conducted a longitudinal serologic survey on a cohort patient who had recovered from the disease in Suzhou, Jiangsu Province, China. We screened 34 patients who had laboratory-confirmed cases and were >18 years of age when they were discharged from the hospital (Figure 1). We enrolled 25 of these patients in our study after obtaining informed consent and prospectively followed them at ≈100, 200, and 300 days after symptom onset (Figure 1). In addition, we enrolled 10 control subjects who live in an area without known H7N9 virus detections, denied close contact with live poultry or live bird markets during the previous 12 months, and had no known diseases or conditions that would reduce their immune response.

We obtained written informed consent from all participants before conducting interviews and sample collection. The study protocol was approved by the Institutional Review Boards of Beijing Institute of Microbiology and Epidemiology and Suzhou Municipal Center for Disease Control and Prevention. The study was also approved by the Animal Care and Use Committee at the Academy of Military Medical Sciences.

### Sampling and Data Collection

At patient enrollment, we used a comprehensive questionnaire to collect information about patients’ demographic and clinical characteristics, history of exposure to poultry, and history of seasonal influenza vaccination. We included archived acute- or convalescent-phase serum samples from the participants in our study. At each of the 3 follow-up visits, we asked each participant to provide a 5-mL blood sample. We used a shorter questionnaire to collect information about demographic characteristics, recent history of exposure to poultry, and experience of influenza-like illness.

### Serologic Testing

We measured serum hemagglutination inhibition (HI) antibody (*8*) by the HI assay; neutralizing antibody by the microneutralization (MN) assay (*9*); neuraminidase inhibition (NI) antibody by the enzyme-linked lectin assay (ELLA) (*10*); and IgG or IgA antibodies by ELISA (*5*). For HI, NI, and MN detection, we applied 2-fold serial dilutions of serum from 1:10 to 1:280. We defined the HI titer as the reciprocal of the highest serum dilution that completely inhibited hemagglutination; the NI titer as the reciprocal of highest serum dilution that exhibited 50% inhibition concentration (IC_50_); and the MN titer as the reciprocal of the highest serum dilution that yielded >50% neutralization. For final titers <1:10 we assigned a value of 1:5 (seronegative). For IgG and IgA detection, we tested serum samples at a starting dilution of 1:50 with 2-fold serial dilutions to 1:12,800. The endpoint titer was the highest dilution giving an optical density at least twice that of background. The final titers <1:50 we assigned a value of 1:25. We used a human H7N9 isolate (A/Jiangsu/Wuxi05/2013) for the HI and MN assays. We used a genetic reassortant H6N9 virus, which contains the hemagglutinin gene of H6N1 virus A/Taiwan/1/2013, the neuraminidase gene of H7N9 virus A/Anhui/1/2013, and other internal genes of A/Puerto Rico/8/1934 H1N1, for ELLA. We used currently circulating human seasonal viruses (A/Shanghai/SWL1970/2015/H1N1 and A/Switzerland/9715293/2013/H3N2) to examine the serum samples for cross-reacting antibodies with HI assay.

We defined a seroprotective threshold as an HI, NI, or MN antibody titer of 40. A titer of >1:40 of HI, NI, or MN antibody has been shown to protect against seasonal influenza viruses (*11*–*13*) and is considered protective against H7N9 infection in humans, but has not been proven so. There is no established correlation of protection for IgG and IgA titers for influenza virus infection, but any detectable antibody level is deemed protective. We set the cutoff value for IgG titer to 1:400 because the mean titer among control serum samples was 1:350.

### Human Serum Passive Transfer and H7N9 Infection of Mice

We obtained 42 female 4-week-old specific pathogen-free BALB/c mice from the Laboratory Animal Center, Academy of Military Medical Sciences, Beijing, China. The mice weighed 13.3 ± 0.9 g. We injected 3 mice per group intravenously with 40 μL of human serum (2-fold serial HI titration ranged from 1:5 to 1:1,280) 12 hours before injecting them intranasally with 20 μL of 10 × 50% lethal dose of H7N9 virus. We gave an equal volume of healthy donor serum or phosphate-buffered saline to control mice. We observed the mice daily for signs of disease for <3 days. We conducted all work with the H7N9 virus in the Biosafety Level 3 laboratory of the State Key Laboratory of Pathogen and Biosecurity.

### Antibody in Serum and Virus Titers in Lungs of Mice

We collected blood from the mice 12 hours after injection with human serum. Because we transferred only 40 μL serum to the mice and there was ≈30-fold dilution of >1 mL blood, we expected the HI titer in mice to be undetectable. Therefore, we used the ELISA method to measure IgG titers. To obtain virus titers, we harvested the lungs of 3 mice at 3 days after virus infection and homogenized them into 1.5 mL of Dulbecco’s Modified Eagle Medium using a manual homogenizer. We aliquoted lung homogenates and kept them at −80°C. We determined the viral titer using the tissue culture infectious dosage on MDCK cells.

### Statistical Analysis

We analyzed the antibody titers with log_10_-transformed geometric means and 95% CIs. We calculated the proportion of antibody titers equal to or greater than seroprotective threshold (HI, NI, and MN) or limit of detection and associated 95% CI. We used Mann-Whitney U test for testing the differences in antibody titers and χ^2^ test and Fisher exact test for testing the differences in proportion of antibody titers above thresholds. All statistical tests were 2-sided with a significance level of 0.05. We conducted all statistical analyses using GraphPad Prism software (GraphPad Software, Inc., La Jolla, CA, USA).

## Results

During October 1, 2016–February 14, 2017, we enrolled in our study 25 laboratory-confirmed H7N9 survivors from Suzhou, Jiangsu Province, China (Figure 1). Among these survivors, 17 were men and 8 were women; the median age was 59 years (range 49.5–66.5 years) (Table 1). All patients required hospitalization at 1–12 days after symptom onset. Most of the patients had severe illness and were admitted to the intensive care unit (ICU). Patients remained in the ICU for 7–30 days. Clinical symptoms included fever, cough, sore throat, fatigue, myalgia, chills, and dyspnea (Table 2). All patients received oseltamivir, and 21 received glucocorticoid for treatment. Laboratory tests at hospital admission showed that some patients had abnormal hepatic function. Most patients had low to medium viral load (Technical Appendix Table 1). In addition, H7N9 viruses isolated from 11/25 patients were of low pathogenicity and belonged to the Yangtze River Delta hemagglutinin lineages (Technical Appendix Table 1). The radiographic findings included pneumonia, increased markings, fuzzy patch lesions, and patch effusion shadows in lungs (Technical Appendix Table 2).

**Table 1:** Clinical characteristics of influenza A(H7N9) virus survivors, China, 2017*

Patient no.	Age, y/sex	Symptoms	Days to admission†	Hospitalization, d	ICU, d	Disease severity
1	89/M	Fever, cough, sore throat, fatigue, myalgia	6	18	16	Severe
2	32/M	Fever, cough, sore throat	12	22	12	Severe
3	41/F	Fever, cough, sore throat, fatigue	8	19	14	Severe
4	83/M	Fever, cough, fatigue	4	11	9	Severe
5	62/M	Fever, cough	7	12	12	Severe
6	71/M	Fever, cough	9	18	13	Severe
7	63/F	Fever, cough, fatigue	9	16	10	Severe
8	54/F	Fever, cough, sore throat	7	14	9	Severe
9	54/F	Fever, cough, fatigue	7	17	17	Mild
10	60/F	Fever, cough, fatigue, chills	7	28	23	Severe
11	28/F	Fever, cough, fatigue	7	16	11	Severe
12	63/M	Fever, cough	6	14	14	Severe
13	65/M	Fever, cough	5	19	0	Severe
14	35/M	Fever, cough, sore throat, fatigue	6	12	12	Severe
15	39/F	Fever, cough	7	19	19	Severe
16	57/M	Fever, cough	10	17	17	Severe
17	75/M	Fever, cough, fatigue	9	22	13	Severe
18	58/M	Fever, cough, myalgia	5	15	8	Mild
19	54/F	Fever, fatigue, myalgia	5	11	11	Severe
20	59/M	Fever, cough	5	21	7	Severe
21	68/M	Fever, cough, dyspnea	5	30	30	Severe
22	59/M	Fever, cough	7	22	22	Severe
23	45/M	Fever, cough	1	13	9	Severe
24	71/M	Fever, cough	2	73	NA	Severe
25	64/M	Fever, cough, sore throat	6	13	11	Mild

*ICU, intensive care unit; NA, not available. †After symptom onset.

**Table 2:** Underlying disease, complications, and treatment of influenza A(H7N9) virus survivors, China, 2017*

Patient no.	Underlying disease	Complications	Oxygen therapy	Mechanical ventilation	Days to antiviral treatment†	Oseltamivir	Glucocorticoid
1	HTN, DM	ARDS, RF	Yes	No	6	Yes	Yes
2	No	ARDS, RF	Yes	No	13	Yes	Yes
3	No	ARDS, RF	Yes	No	8	Yes	Yes
4	COPD, HTN	No	Yes	No	6	Yes	Yes
5	HTN	ARDS, RF	Yes	No	7	Yes	Yes
6	HTN, DM	ARDS, HI	Yes	No	14	Yes	No
7	No	ARDS	Yes	No	9	Yes	Yes
8	No	No	Yes	No	7	Yes	Yes
9	No	No	Yes	No	9	Yes	Yes
10	No	ARDS, RF, HI, RI	Yes	Yes	11	Yes	Yes
11	No	HI	Yes	No	7	Yes	Yes
12	No	No	Yes	No	8	Yes	No
13	No	No	Yes	No	8	Yes	Yes
14	No	No	Yes	No	6	Yes	Yes
15	No	HI	Yes	No	7	Yes	Yes
16	No	RF	Yes	No	13	Yes	No
17	No	ARDS	Yes	No	9	Yes	Yes
18	No	No	Yes	No	13	Yes	Yes
19	No	RF, HI	Yes	No	5	Yes	Yes
20	HTN	ARDS, RF, HI	Yes	Yes	11	Yes	Yes
21	HTN, DM, HL	ARDS, RF, HI	Yes	Yes	5	Yes	Yes
22	HTN	ARDS, RF, HI	Yes	No	7	Yes	Yes
23	HTN	No	Yes	No	6	Yes	NA
24	NA	NA	Yes	NA	1	Yes	NA
25	CHB	HI	Yes	No	9	Yes	Yes

*ARDS, acute respiratory distress syndrome; CB, chronic bronchitis; CHB, chronic hepatitis B infection; COPD, chronic obstructive pulmonary disease; DM, diabetes mellitus; HI, hepatic insufficiency; HL, hyperlipidemia; HTN, hypertension; NA, not available; RF, respiratory failure; RI, renal insufficiency. †After admission.

Table 3 shows the proportion of survivors with antibody titers equal to or greater than the seroprotective threshold (1:40 for HI, NI, and MN) or the minimum detection limit (1:400 for IgG and 1:50 for IgA) at each time point. Counting from the day of symptom onset, >90% of survivors had an HI titer >1:40 on day 100. This proportion reached 82.6% on day 200 but decreased to 36.4% on day 300. The overall patterns of the NI antibody titers were similar to the HI antibody titers, except that 63.6% of survivors had an NI titer >1:40 on day 300. Unlike the HI and NI antibody titers, the proportion of seroprotective MN (≈86%) and IgG (100%) titers remained steady over time. For IgA antibody titers, the seroprotective proportion decreased from 96% on day 100 to ≈60% on day 300, an absolute reduction of >30%.

**Table 3:** Proportion of influenza A(H7N9) virus survivors with titers at seroprotective levels at acute phase of infection and 3 follow-up points after infection, China, 2017*

Antibody	% Patients (95% CI)			
Acute phase	Follow-up visit 1	Follow-up visit 2	Follow-up visit 3
HI	54.5 (32.2–75.6)	92.0 (74.0–99.0)	82.6 (61.2–95.0)	36.4 (17.2–59.3)
NI	50.0 (28.2–71.8)	96.0 (79.6–99.9)	91.3 (72.0–98.9)	63.6 (40.7–82.8)
MN	22.7 (7.8–45.4)	88.0 (68.8–97.5)	87.0 (66.4–97.2)	86.4 (65.1–97.1)
IgG	45.5 (24.4–67.8)	100 (86.3–100)	100 (85.2–100)	100 (84.6–100)
IgA	54.5 (32.2–75.6)	96.0 (79.6–99.9)	60.9 (38.5–80.3)	59.1 (36.4–79.3)

*Seroprotective levels for HI, NI, and MN titers, >1:40; IgG >1:400; or IgA >1:50. HI, hemagglutination inhibition; MN, microneutralization; NI, neuraminidase inhibition.

**Figure 2:**
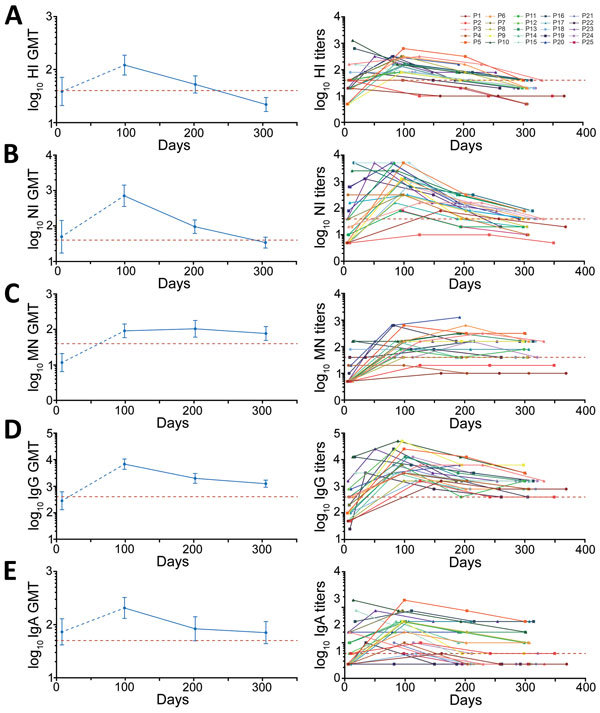
GMTs (left) and individual titers (right) of antibodies to influenza A(H7N9) virus in serum samples collected from survivors, China, 2017: A) HI, B) NI, C) MN, D) IgG, and E) IgA. Red dashed line indicates threshold for seroprotective titer (HI, NI, and MN = 1:40) or limited detection titer (IgG = 1:400; IgA = 1:50). Error bars indicate 95% CIs. GMT, geometric mean titer; HI, hemagglutination inhibition; MN, microneutralization; NI, neuraminidase inhibition; P, patient.

The geometric mean titers (GMTs) of antibodies were plotted by the time points in Figure 2. Overall, ≈300 days after symptom onset, HI and NI GMTs substantially declined and were lower than the seroprotective threshold of 1:40 and the GMTs in the acute phase (Figure 2, panels A and B). In contrast, the MN GMTs increased over time, peaked on day 200, and then declined by day 300, yet remained considerably above the GMTs in the acute phase and the seroprotective threshold of 1:40 (Figure 2, panel C). Although we observed no substantial difference in GMTs across 3 follow-up time points, the MN GMTs on day 200 were relatively high, suggesting a possible delayed response after infection. IgG and IgA decreased gradually from day 100 to day 300 but remained higher than the limit of detection (Figure 2, panel D). However, IgG GMTs on day 200 and day 300 were substantially higher than the IgG GMTs in the acute phase, whereas the IgA GMTs on day 200 and day 300 were similar to those in the acute phase. There were no detectable antibodies to the H7N9 virus in the control subjects, but GMT was 283.3 (titer ranged from 1:200 to 1:800) for IgG, suggesting a possible cross-reactivity between the H7N9 virus and other subtypes.

Approximately 300 days after symptom onset, nearly all survivors had a >4-fold decline in the HI titer compared with the titer on day 100, and 14 survivors had HI titers <1:40 (Table 4,5; Figure 2, panel A). Among these 14 survivors, 2 (patients 1 and 2) maintained low titers (1:10) throughout the study period, but 2 others (patients 4 and 25) had undetectable titers around day 300. The other 10 survivors had titers of 1:20. Twenty-one survivors had a >4-fold decrease of the NI titer ≈300 days after symptom onset, and 8 survivors (patients 1, 2, 4, 8, 11, 14, 22, and 25) had titers <1:40 (Table 4,5). Among these 8 survivors, the titer of patient 2 declined to seronegative on day 349, and others had titers <1:20 (Table 4,5; Figure 2, panel B). In contrast, the majority of survivors had a >2-fold increase in MN titer (9 survivors) or maintained MN titer (8 survivors) on day 200 in comparison to day 100 after symptom onset, followed by a decrease or a maintenance on day 300 (Figure 2, panel C; Table 4,5). However, 6 survivors (patients 1, 2, 4, 8, 21, and 25) maintained low titers over the study period (Table 4,5; Figure 2, panel C), but none of them became seronegative. Although most of the survivors had a ≥4-fold decline in IgG titer over time, all survivors maintained detectable antibody titers ≥1:400 (Table 4,5; Figure 2, panel D). However, the overall response of IgA antibody was relatively weak, and 9 survivors (patients 1, 3, 4, 15, 18, 19, 21, 22, and 25) already had undetectable titer on day 200 (Tables 4, 5; Figure 2, panel E).

**Table 4:** Antibody titers in survivors of influenza A(H7N9) during the acute phase and at 3 follow-up points, China, 2017*

Patient no.	HI/NI/MN/IgG/IgA titers			
Acute phase	Follow-up visit 1	Follow-up visit 2	Follow-up visit 3
1	20/5/5/50/25	10/80/10/1600/50	10/40/10/800/25	10/20/10/800/25
2	20/5/5/50/50	10/10/20/1600/100	10/10/20/400/50	10/5/20/400/50
3	160/20/5/400/200	320/160/160/6400/50	160/160/320/6400/25	40/40/160/1600/25
4	40/320/20/400/25	40/320/20/3200/100	20/40/10/1600/25	5/10/10/800/25
5	5/5/5/100/25	640/5120/640/25600/1600	320/320/320/12800/800	40/80/320/3200/400
6	20/20/5/400/50	320/1280/160/3200/200	160/160/640/800/100	20/40/160/800/100
7	40/40/5/200/200	320/320/160/6400/800	NA	NA
8	40/5/5/50/25	320/320/40/1600/400	80/80/40/1600/200	20/20/40/800/100
9	5/5/5/100/25	80/1280/160/51200/400	40/40/160/6400/50	40/40/160/6400/50
10	1280/2560/160/12800/1600	160/2560/160/51200/800	80/320/320/12800/400	40/80/320/3200/200
11	40/10/5/100/100	80/80/80/6400/400	40/20/40/400/200	20/20/80/1600/100
12	40/40/5/800/50	80/5120/40/25600/200	NA	NA
13	20/10/5/400/100	160/1280/160/12800/400	80/320/320/3200/200	40/80/160/1600/200
14	5/5/5/50/25	160/160/160/3200/400	40/40/40/800/100	20/20/40/800/100
15	160/5120/160/3200/800	80/5120/160/3200/200	40/80/160/1600/25	20/80/160/1600/25
16	640/5120/160/12800/400	160/640/80/6400/800	80/320/160/3200/400	40/80/160/1600/400
17	20/160/5/100/25	160/320/80/12800/400	80/80/80/1600/400	40/40/80/1600/400
18	80/5/80/100/25	80/1280/80/1600/50	40/80/160/800/25	20/40/160/800/25
19	40/80/10/200/25	320/2560/640/25600/25	40/160/160/1600/25	20/40/160/1600/25
20	40/640/20/800/200	320/5120/640/3200/200	160/160/1280/6400/200	NA
21	NA	160/5120/40/6400/100	40/160/40/1600/25	20/80/40/1600/25
22	20/640/40/25/25	80/1280/40/3200/100	40/320/80/800/25	20/20/40/400/25
23	20/40/5/1600/200	320/5120/160/25600/800	80/160/160/3200/400	80/80/160/1600/400
24	NA	80/640/40/12800/100	40/160/160/3200/50	20/40/40/1600/50
25	NA	40/80/40/3200/50	20/20/40/1600/25	5/10/40/400/25

*HI, hemagglutination inhibition; MN, microneutralization; NA, not available; NI, neuraminidase inhibition.

**Table 5:** Change in antibody titers in survivors of influenza A(H7N9) at different follow-up points, China, 2017*

Patient no.	Change, -fold, HI/NI/MN/IgG/IgA	
	Follow-up visit 2 vs. follow-up visit 1	Follow-up visit 3 vs. follow-up visit 2
1	1/0.5/1/0.5/0.5	1/0.5/1/1/1
2	1/1/1/0.25/0.5	1/0.5/1/1/1
3	0.5/1/2/1/0.5	0.25/0.25/0.5/0.25/1
4	0.5/0.13/0.5/0.5/0.25	0.25/0.25/1/0.5/1
5	0.5/0.06/0.5/0.5/0.5	0.13/0.25/1/0.25/0.5
6	0.5/0.13/4/0.25/0.5	0.13/0.25/0.25/1/1
7	NA	NA
8	0.25/0.25/1/1/0.5	0.25/0.25/1/0.5/0.5
9	0.5/0.03/1/0.13/0.13	1/1/1/1/1
10	0.5/0.13/2/0.25/0.5	0.5/0.25/1/0.25/0.5
11	0.5/0.25/0.5/0.06/0.5	0.5/1/2/4/0.5
12	NA	NA
13	0.5/0.25/2/0.25/0.5	0.5/0.25/0.5/0.5/1
14	0.25/0.25/0.25/0.25/0.25	0.5/0.5/1/1/1
15	0.5/0.02/1/0.5/0.125	0.5/1/1/1/1
16	0.5/0.5/2/0.5/0.5	0.5/0.25/1/0.5/1
17	0.5/0.25/1/0.13/1	0.5/0.5/1/1/1
18	0.5/0.06/2/0.5/0.5	0.5/0.5/1/1/1
19	0.13/0.06/0.25/0.06/1	0.5/0.25/1/1/1
20	0.5/0.03/2/2/1	NA
21	0.25/0.03/1/0.25/0.25	0.5/0.5/1/1/1
22	0.5/0.25/2/0.25/0.25	0.5/0.06/0.5/0.5/1
23	0.25/0.03/1/0.13/0.5	1/0.5/1/0.5/1
24	0.5/0.25/4/0.25/0.5	0.5/0.25/0.25/0.5/1
25	0.5/0.25/1/0.5/0.5	0.25/0.5/1/0.25/1

*Change was calculated as ratio of titers. HI, hemagglutination inhibition; MN, microneutralization; NA, not available; NI, neuraminidase inhibition.

**Figure 3:**
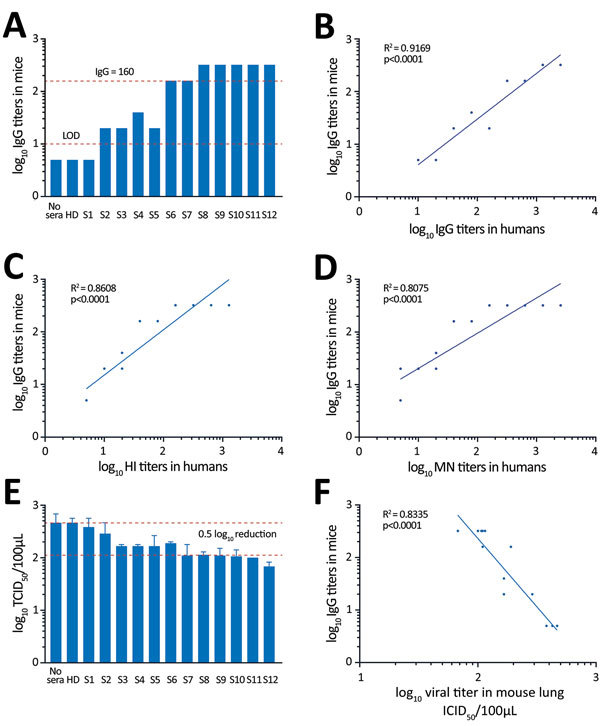
Testing of convalescent-phase serum transfer as potential protection against influenza A(H7N9) virus infection. Mice received 40 μL of patient serum intravenously 12 hours before H7N9 virus infection. A) IgG titers from mouse serum samples collected 1 h before infection. B–D) Relationships between IgG, HI, and MN titers in human serum and IgG titer in mouse recipients of transferred serum. E) Virus titers in homogenized mouse lungs at day 3 after infection (mean ± SE). F) Relationship between IgG titer in mouse serum samples and viral titers in mouse lung samples. HD, healthy donor; HI, hemagglutination inhibition; LOD, limit of detection; MN, microneutralization; S, serum; TCID_50_, 50% tissue culture infectious dose.

To further assess the physiologic contribution of the magnitude of the HI antibody titers, we transferred 40 μL convalescent-phase serum from individual patients to mice (Technical Appendix Table 3). IgG titers in the serum samples of recipient mice correlated well with IgG, HI, and MN titers in the human serum samples, but we observed a better correlation for IgG titer in the human samples (Figure 3, panels A–D). Mouse IgG titers in the serum samples at the time of challenge correlated inversely with virus titers in the lung samples, confirming the importance of neutralizing antibodies assessed in laboratory analysis in virus clearance (Figure 3, panels E, F). These results also suggest that an IgG titer of >1:160 was required to reduce virus titers by 0.5 log_10_ in infected mice. Assuming that these numbers can be extrapolated to patients, transferring 40 μL of serum to a 13-g mouse is equivalent to transferring 210 mL of serum to a 70-kg patient (calculated per kilogram), thereby providing a potential guideline for its use in clinical settings.

The different types of antibody measures are significantly correlated with each other; we observed higher correlation between HI and NI and between NI and IgG at R^2^ >0.5 (Technical Appendix Figure 1). We found no correlation between antibodies to the H7N9 virus and HI antibodies against seasonal H1N1 and H3N2 viruses (p>0.05) (Technical Appendix Figure 2), indicating that there is no heterologous boost of antibodies against H7 by H3 or H1 hemagglutinin. The antibody responses did not vary by patient age, sex, presence of underlying conditions, time in ICU, ventilation, or disease severity.

## Discussion

In our study, antibodies to H7N9 virus waned over time, but most survivors maintained detectable antibody titers ≈1 year after infection. However, >60% of survivors had an HI titer <1:40, which is potentially not seroprotective, ≈300 days after infection. Antibody responses were highly variable in survivors, and a few of them had weak antibody responses or had quickly waning antibody titers that were undetectable ≈1 year after infection despite their severity of infection. We also identified a threshold of IgG titer that was crucial to virus clearance in the animal model and could be useful in clinical settings.

HI antibodies induced by natural infection with the 2009 pandemic H1N1 virus persist at constant high titer (>1:40) for a minimum of 15 months (*14*). Additionally, the HI antibody against the H5N1 virus infection is reported to last even longer, at a stable titer (≥1:40) for nearly 5 years, although only a few survivors have been studied (*15*). In contrast, our study shows that only 36.4% of H7N9 survivors had HI titers >1:40 at ≈1 year after infection, although most survivors had detectable HI antibody titers. On the other hand, we observed relatively high MN antibody titers persisting over time in survivors, and these levels were sufficient to predict protection, based on the protection extrapolated from seasonal influenza. If we assume that MN antibody is truly a better correlate of protection than HI antibody and a titer of >1:40 is sufficient for protection, we could anticipate that most H7N9 survivors would remain protected against the H7N9 virus >1 year after infection.

It has been observed that antibody responses in infections with H5N1 or 2009 pandemic H1N1 virus in which patients had mild or no symptoms waned faster than those in patients with severe influenza disease and decreased below the threshold of positivity within 1 year (*16*,*17*). Of all reported H7N9 cases, <10% were asymptomatic or mild (*18*–*20*), and our study included only 3 mildly symptomatic patients (patients 9, 18, and 25). These 3 patients maintained detectable NI, MN, and IgG antibodies, but patient 25 became seronegative for HI antibodies and IgA on or around day 305 after symptom onset. Meanwhile, several severely ill patients had relatively weak antibody responses 100 days after symptom onset; in particular, patients 1, 2, and 4 either maintained low antibody titers over time or became seronegative at ≈1 year after symptom onset. Therefore, there was no clear association between disease severity and antibody response. Nevertheless, most patients in our study had severe symptoms, and so the findings may not be representative of mild cases or asymptomatic infections.

Previous seroepidemiological studies have identified the subclinical infections among both occupationally exposed workers and the general population, but the results varied (*21*–*29*). Similar to the problems with H5N1 infections in humans (*30*), the serologic threshold titer to recognize subclinical infections of the H7N9 virus is not yet established, which leads to difficulty in estimating the seroprevalence of subclinical infections. A major problem in identifying such a serologic threshold for seropositivity is the insufficient immunogenicity of the H7 hemagglutinin (*31*–*34*). Our results show that HI, NI, IgG, and IgA antibodies declined substantially over time. In particular, for HI antibody, >60% of survivors had a titer <1:40, and 2 of them became negative at ≈1 year after infection. Given the low magnitude in HI antibody response, the true incidence of H7N9 infection is likely to be underestimated if a titer of >1:40 is used as the serologic threshold for the HI assay in seroepidemiological studies. In our study, the level of MN antibody titer was relatively stable over time and correlated well with other types of antibodies; therefore, the MN antibody could be a more useful indicator than HI for determining the incidence of infection.

Convalescent plasma therapy has been considered as a treatment option for new and emerging infectious diseases for which effective drugs and vaccines are not readily available. Although the anti–influenza A virus drug oseltamivir appeared to be useful for the treatment of H7N9 infection, the ≈40% mortality rate still remains a challenge for clinical treatment, especially for severe cases who visit a hospital days past onset of their symptoms. Previous studies have shown that convalescent plasma treatment reduced the mortality rate of severe 2009 pandemic H1N1 infection (*35*) and benefited patients with severe H5N1 or H7N9 infections (*36*,*37*). Our results indicate that, despite substantially decreased HI antibody titers over time, most of the survivors still had a titer >1:40 ≈200 days after infection, and high antibody titers are likely for other antibodies from survivors’ serum. The experimental results from our model using mice suggest that transferring 210 mL of serum with HI titer >1:80 to a 70-kg patient is a possible guideline for clinical treatment.

Our study had some limitations. First, our results need to be validated with larger numbers of survivors. We have thus far included 25 survivors, ≈3% of all reported H7N9 survivors in China. The small sample size limited our ability to analyze the antibody response stratified by patient age, sex, underlying condition, or disease severity. Second, we could not collect blood samples more frequently from the patients, especially between acute phase and ≈100 days after infection, which could have provided a more complete picture about the dynamics of antibody responses. Finally, we did not study the virus-specific memory T- and B-cell response because of constraints in logistics. Whether there are correlations between cellular immune responses and antibody responses needs further investigation.

In conclusion, our findings contribute to the understanding of individual immune responses to H7N9 virus infection and of population-based immunity in regions where H7N9 virus outbreaks have occurred. Our study provides a useful serologic guideline for developing effective vaccines and therapies to counter H7N9 virus infections.

Technical Appendix Additional information about influenza A(H7N9) virus antibody responses in survivors 1 year after infection, China.
