# Supplementary material for: Influenza A(H7N9) Virus Antibody Responses in Survivors 1 Year after Infection, China, 2017
Source: Emerg Infect Dis. 2018 Apr;24(4):663–72. doi: 10.3201/eid2404.171995 (PMC5875250; doi:10.3201/eid2404.171995)
Supplement: Technical Appendix — Additional information about influenza A(H7N9) virus antibody responses in survivors 1 year after infection, China. [file 17-1995-Techapp-s1.pdf]

Article DOI: https://doi.org/10.3201/eid2404.171995

Influenza A(H7N9) Virus Antibody Responses in Survivors 1 Year after Infection, China, 2017

Technical Appendix

Technical Appendix Table 1. Laboratory data for patients infected with influenza A(H7N9) virus on hospital admission, China, 2017*

Patient	C_t†							Creat,	Leukocyte,			
no.	Flu A	H7	N9	Virus isolation	HA lineage	ALT, U/L	AST, U/L	μmol/L	$\times 10^9$/L	CRP, mg/L	Temp, °C	Serum collection, d§
1	32.54	29.04	30.35	Yes	YRD†	23	31	60.9	4.41	29.8	39	6, 161, 258, 369
2	27.56	32.64	30.89	No	NA	63	41	50.6	9.7	13	38	10, 126, 242, 349
3	29.37	28.03	29.32	No	NA	31	34	34.8	5.2	35.7	39.5	8, 126, 230, 332
4	30.58	32.96	30.79	No	NA	63	54	72.2	4.1	22.5	39.3	7, 99, 204, 307
5	30.04	29.97	30.72	Yes	YRD†	34.3	141.9	49	2.7	15.3	39	5, 99, 203, 300
6	28.12	29.35	28.58	No	NA	103	242	76	4.51	14.85	38.6	14, 99, 202, 307
7	28.62	28.25	28.54	No	NA	19	28	38.1	4.2	22.7	38.5	8, 96
8	30.12	29.57	29.51	Yes	YRD‡	113	118	70.8	4.12	197	40.5	7, 99, 203, 305
9	28.45	28.71	28.56	Yes	YRD‡	21	61	55	2.53	63.5	42	7, 96, 195, 298
10	30.56	31.63	31.95	No	NA	36	24	92	4.67	54.6	39.9	14, 89, 193, 301
11	28.52	29.13	29.65	Yes	YRD‡	15	16	50.6	3.59	31	39.9	7, 94, 194, 299
12	27.43	28.57	27.78	Yes	YRD‡	119.7	142.1	86	7.76	113.92	39.5	7, 84
13	30.5	30.28	32.1	Yes	YRD‡	219	53	64.5	4.1	67	39	8, 101, 189, 300
14	27.19	28.04	27.67	Yes	YRD‡	21	54	108.6	4.55	70	40.5	6, 85, 189, 298
15	28.55	29.01	28.69	Yes	YRD‡	45.7	56	33	4.92	15.42	40	19, 109, 214, 310
16	30.67	28.85	30.88	No	NA	33.5	80.5	57	4.92	14.68	39.5	17, 111, 215, 314

Patient	C_t†							Creat,	Leukocyte,			
no.	Flu A	H7	N9	Virus isolation	HA lineage	ALT, U/L	AST, U/L	μmol/L	$\times 10^9$/L	CRP, mg/L	Temp, °C	Serum collection, d§
17	28	28.45	29.51	Yes	YRD‡	39	54	61.5	15.7	86.3	39	10, 106, 210, 307
18	28.18	28.36	29.05	No	NA	54	40	63.1	4.3	13	39.6	10, 119, 209, 318
19	30.22	29.75	30.52	No	NA	46.3	78.5	52	2.75	13.33	39	8, 82, 186, 292
20	29.35	29.24	29.02	No	NA	210	157	84	2.3	115	40	11, 79, 192
21	NA	NA	NA	NA	NA	NA	NA	NA	NA	NA	39	82, 186, 285
22	27.15	27.03	27.13	No	NA	32.3	89.3	92	2.49	15.92	39.6	35, 149, 261
23	27.67	28.87	28.73	No	NA	55	51	52	5.9	22.4	39.8	9, 51, 147, 254
24	NA	NA	NA	No	NA	NA	NA	NA	3.2	NA	40	6, 114, 211, 321
25	29.03	28.45	29.64	Yes	YRD‡	34	67	60.8	3.36	92.9	39	98, 195, 305

*ALT, alanine transaminase; AST, aspartate transaminase; Creat, creatinine; CRP, C-reactive protein; Ct, cycle threshold; leukocyte, leukocyte (leuckocyte) count; NA, not available; temp, body temperature; YRD, Yangtze River Delta lineage.

†Ct values are based on virus levels detected in throat swabs of the patients.

‡Low pathogenicity avian influenza virus.

§After illness onset.

Technical Appendix Figure Table 2. Radiographic findings for patients infected with influenza A(H7N9) virus, China, 2017*

Patient no.	Radiographic findings
1	Inflammation in both lungs
2	Inflammation in both lungs
3	Exudative lesions in both lungs, inflammation in left lung
4	Infection in both lungs
5	Infection in left lung, left pleural effusion
6	Infection in both lungs, pleural thickening in both sides, left pleural effusion
7	Inflammation in both lungs
8	Infection, consolidation, and effusion in both lungs
9	Inflammation in both lungs
10	Inflammation in both lungs
11	Multi-patchy high-density shadow in both lungs
12	Inflammation in both lungs
13	Infection and consolidation in left lung
14	Infection and interstitial inflammation in right lower lung
15	Large high-density shadow and consolidation in two lungs
16	Infection in two lungs
17	Pneumonia in two lungs and particularly the right lung
18	Pneumonia in right lower lung
19	Ground-glass opacity in 2 lungs
20	Inflection, emphysema, bullae in 2 lungs. Hydropneumothorax in right lung, effusion in left lung
21	NA
22	Consolidation in right lower lung. Hydropneumothorax with pulmonary atelectasis in right lung
23	Pneumonia in right lower lung
24	NA
25	Infection in right lower lung and right pleural effusion

*NA, not available.

Technical Appendix Table 3. Antibody titers in human and mouse serum, and virus titer in mice lungs

Serum no.	IgG antibody of human serum	HAI antibody of human serum	MN antibody of human serum	IgG antibody titer in mouse serum	$TCID_{50}$ in mice lungs
S1	200	5	5	5	2.58
S2	400	10	5	20	2.46
S3	1600	20	10	20	2.22
S4	800	20	20	40	2.22
S5	1600	20	20	20	2.22
S6	3200	40	40	160	2.28
S7	6400	80	80	160	2.04
S8	12800	160	160	320	2.06
S9	25600	160	640	320	2.04
S10	12800	320	320	320	2.03
S11	12800	640	1280	320	2.00
S12	12800	1280	2560	320	1.83
PBS	—	—	—	5	2.67
HD	100	5	5	5	2.63

*HD, healthy donor; HAI, hemagglutination inhibition; MN, microneutralization; S, serum.

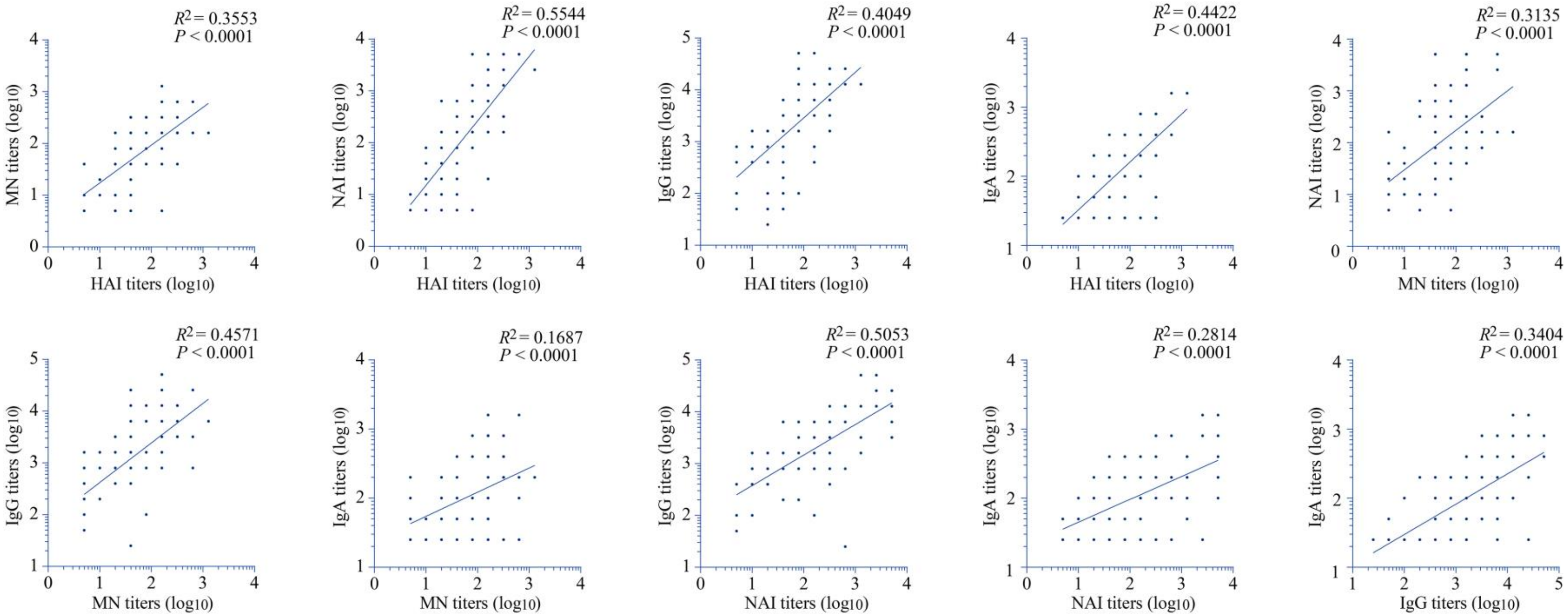

Technical Appendix Figure 1. Correlation analysis among titers of hemagglutination inhibition (HI), neuraminidase inhibition (NI), microneutralization (MN), IgG, and IgA antibodies against the influenza A(H7N9) virus in survivors.

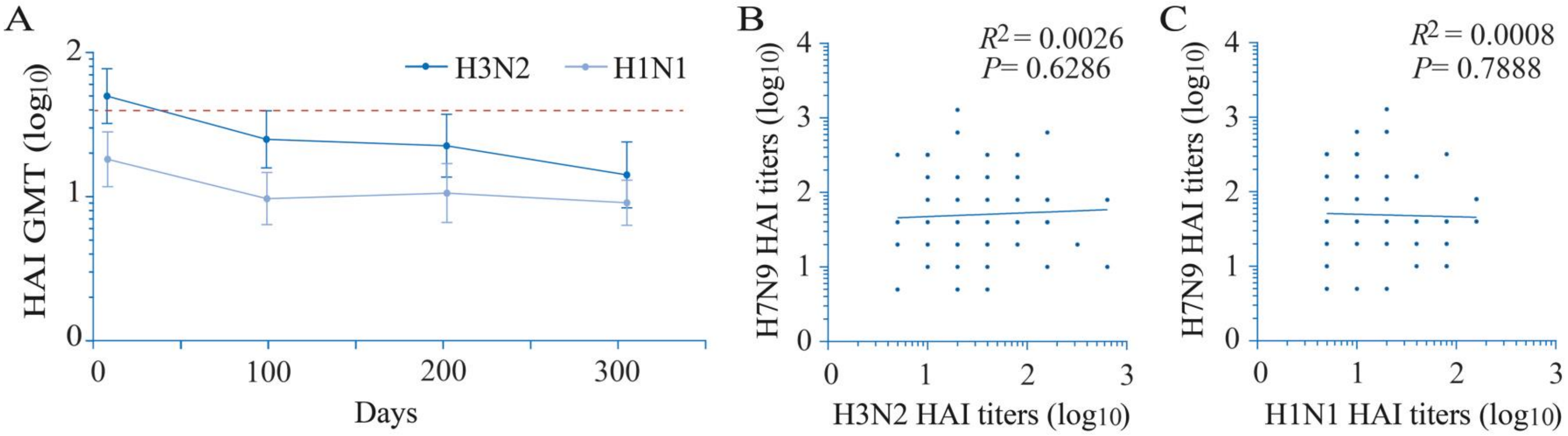

Technical Appendix Figure 2. Hemagglutination inhibition (HI) antibodies to human seasonal influenza H3 and H1 viruses in survivors and their correlation with HI antibody to the H7N9 virus. A) HI geometric mean titer (GMT) to H1 and H3 subtypes in influenza survivors. B) Correlation between HI antibody to the H7N9 virus and H3 (B) subtype. C) Correlation between HI antibody to the H7N9 virus and H1 subtype.
